# Factors Associated With Poor Sleep Quality Among Primary Healthcare Workers During the SARS-CoV-2 Pandemic

**DOI:** 10.7759/cureus.56502

**Published:** 2024-03-19

**Authors:** Jorge I Zurutuza, Liliana Ovando-Diego, Marco A Lezama-Prieto, Jaime Morales-Romero, Montserrat A Melgarejo-Gutierrez, Christian S Ortiz-Chacha

**Affiliations:** 1 Epidemiology and Biostatistics Department, Centro de Investigaciones Biomedicas, Xalapa, MEX; 2 Family Medicine Unit No. 66, Instituto Mexicano del Seguro Social, Xalapa, MEX; 3 Family Medicine Unit No. 27, Instituto Mexicano del Seguro Social, Papantla, MEX; 4 Public Health Department, Universidad Veracruzana, Xalapa, MEX; 5 Faculty of Medicine, Universidad Veracruzana, Xalapa, MEX; 6 Public Health Department, Instituto Nacional de Salud Publica, Xalapa, MEX

**Keywords:** covid-19-related mental issues, job stress, primary care physicians, health personnel, pittsburgh sleep quality index (psqi)

## Abstract

Introduction: Sleep is one of the most important activities for health and the processes related to the central nervous system. Healthcare workers commonly present alterations in the sleep-wake cycle due to complex work schedules because 24-hour attention to the population is required in public health institutions. The increase in care needs caused by the COVID-19 pandemic caused changes in work schedules; as in Mexico, the number of patients requiring consultation in all public health units increased. Chronic partial sleep deprivation (< 7 hours of sleep in the 24-hour cycle) is the most frequent sleep alteration in Mexican health workers. However, it has not been explored whether work modifications due to the pandemic had an impact on the sleep quality of workers.

Objective: We aimed to describe the prevalence of poor sleep quality and the associated factors in workers (clinical and non-clinical) of a primary care medical unit.

Material and methods: We conducted an analytical and cross-sectional study during November and December 2022. We used the following tools for studying clinical and non-clinical staff working at a family medicine primary care unit: Pittsburgh Sleep Quality Index, Hamilton Anxiety Scale, Beck Depression Inventory, Maslach Burnout Inventory, and Graffar-Méndez-Castellanos socioeconomic level scale, as well as a data collection sheet and a survey of workers' knowledge, attitudes, fears, and needs regarding COVID-19.

Results: A total of 233 workers were surveyed. The prevalence of poor sleep quality was 56.7%. A higher score on the Beck Depression Inventory (OR: 1.21, CI 95%: 1.13-1.29), being a doctor (OR: 3.48, CI 95%: 1.5-8.01), and frequent alcohol consumption (OR: 2.4, CI 95%: 1.13-5.2) were identified as risk factors for poor sleep quality. A lower score in the depersonalization dimension of the Maslach Burnout Inventory (OR: 0.5, CI 95%: 0.26-0.99) was identified as a protective factor for poor sleep quality.

Conclusions: During the pandemic, the stress of health workers increased due to work alterations that were necessary to treat the greatest number of patients, so their quality of sleep decreased. Unfortunately, the mental health of healthcare workers is often under-assessed in many institutions. Thus, it is relevant to identify risk factors for alterations (especially those of sleep), since by identifying the target population, comprehensive interventions can be carried out, which can reduce the prevalence of burnout, anxiety, and depression, but if not addressed, the alterations can lead to inadequate care for users of health units.

## Introduction

Sleep is a key physiological state controlled by the most important circadian rhythm, being a fundamental part of the sleep-wake cycle. It covers one-third of human life and has an important effect on health [1]. Additionally, it is characterized by a reduction in alertness and a reduction in physiological and cognitive activity accompanied by muscle relaxation [2]. During this phase, new synaptic connections are formed, which are essential for learning, the proper functioning of the executive functions of the brain, and the conservation of energy [3].

Poor sleep quality can occur most frequently due to chronic partial sleep deprivation (CPSD), which is defined as sleeping less than seven hours in the 24-hour cycle. Other less common causes of poor sleep quality are alterations in sleep initiation and/or termination, reduction in latency, sleep efficiency, and awakening status conditions [4,5].

If the quality of sleep is altered, it can lead to various pathological conditions at a behavioral and physiological level, such as alterations in the control of emotions, learning, memory, attention, and planning; increased blood pressure; insulin resistance; and increased production of thyroid-stimulating hormone and cortisol [6,7]. Thus, individuals exposed to poor sleep quality have a higher risk of developing diabetes, hypertension, obesity, and affective disorders, such as depression and anxiety, loss of attention, and increased perceptual and cognitive distortions [7-11].

Health personnel daily experience changes in sleep-wake balance and tend to suffer from CPSD caused by excessive work demand and its complexity and irregular work shift schedules that can be morning, afternoon, or night; discontinuous, semi-continuous, or continuous (those that exceed eight hours); and permanent or rotating shifts, among others [12,13].

The coronavirus-19 disease (COVID-19), caused by a same-named virus, was a spontaneous and relatively unforeseen event that forced a rapid change in the Mexican health system. For workers, it implied an increase in the number of patients to be treated, work hours, and stress, which were both causes and effects of CPSD [14].

The COVID-19 pandemic affected the Mexican health system, increasing the demand for a system with a large overload. This forced a rapid restructuring process called "hospital conversion," which sought to increase care for patients with COVID-19 [15]. These modifications did not include the massive hiring of more personnel, which forced an increase in work overload and stress on the staff.

Therefore, the Mexican Social Security Institute (or IMSS, per its Spanish acronym for Instituto Mexicano del Seguro Social), being one of the largest public care institutions in Mexico, established care guidelines for the mental health of workers, with special emphasis on first-level units known as Family Medicine Units (or UMF, per its Spanish acronym for Unidad de Medicina Familiar) [16]. However, neither the impact of the interventions nor the sleep quality conditions of the workers were measured before and after implementing these care actions.

In this context, our study aimed to describe the prevalence of poor sleep quality and the factors associated with it, in workers (clinical and non-clinical) of a primary care medical unit (or UMF), located in Xalapa, Veracruz, Mexico, during the coronavirus pandemic.

## Materials and methods

Study design

An analytical cross-sectional study was conducted on workers from a public medical unit at the first level of care located in Xalapa, Mexico. From November to December 2022, the instruments were applied, and the collection record was carried out through a face-to-face interview. The personnel who provide clinical care (doctors and nurses) and non-clinical personnel in intermittent contact with patients (nutritionists, social workers, laboratory technicians, psychologists, and administrative personnel) were included, with a work experience of ≥ 6 months in the medical unit. Personnel who worked simultaneously in another health institution and those who did not answer the instruments used or lied about verifiable data were excluded. No sample size calculation was carried out since all workers who met the selection criteria were included; for this same reason, no sampling technique was required. The protocol was approved by the Research and Research Ethics Committees of IMSS, with registration number R-2022-3007-031.

Questionaries and variables

The main variable of the study was sleep quality, measured by the Pittsburgh Sleep Quality Index (PSQI) [17]. The Hamilton Anxiety Scale (HAS), the Beck Depression Inventory (BDI), and the Maslach Burnout Inventory (MBOI) were used to investigate other covariates, such as anxiety, depression, and burnout syndrome, respectively [18-20]. The Graffar-Méndez-Castellanos Socioeconomic Level (GMC-SLE) scale was also applied, as well as a survey that collected information about anthropometric data, sociodemographic characteristics, clinical history, and family structure. Family structure was classified based on the "Basic Concepts for the Study of the Family" from the Mexican Academic Consensus in Family Medicine, in which various health institutions and organizations took part and was carried out in 2005 [21,22]. Additionally, a survey of 19 Likert-type questions was applied that investigated the knowledge, attitudes, fears, and needs of workers regarding COVID-19. The questions asked in this last survey were evaluated independently, and no score was generated.

Poor sleep quality was determined when the PSQI score was ≥ 6 points; the presence of anxiety was defined as a HAS score of > 5 points; and depression was determined when a score was > 14 points in the BDI. Concerning MBOI, each domain has a cohort point: the personal exhaustion domain was considered to have a score > 18 points; the depersonalization domain was considered to have a score of 6 points; and the personal achievement domain was considered to have a score of 33 points or less [17-20]. In the MBOI, the alteration of a single domain implies the presence of this condition; the more domains that are affected, the more serious the alteration.

Statistics analysis

The analysis was performed using, for continuous variables, the Student's t-test for independent groups or the Mann-Whitney U test and, for categorical variables, the chi-square test. A correlation analysis was included using the Spearman test (S) for the instrument scores. Finally, a binary logistic regression model was performed, the dependent variable for which was poor sleep quality (PSQI score ≥ 6), and the independent variables were age, the score on the MBOI depersonalization domain, job category, frequent alcohol consumption, and the presence or absence of some degree of anxiety (cut-off point: 6 points or more on the HAS). Statistical analysis was performed using Statistical Product and Service Solutions (SPSS, version 29; IBM SPSS Statistics for Windows, Armonk, NY) software.

## Results

A total of 233 (100%) clinical and non-clinical workers from all areas of the study unit were included. No subject required exclusion, and there was no dropout in the study. Of the included subjects, 54.5% (127 workers) were women, and 45.5% (106 workers) were men. The average age was 41 ± 8.6 years, the work seniority on IMSS was 16 ± 2.4 months, and the work seniority on UMF was 9 ± 1.8 months. Additionally, 91.4% (213 workers) were permanent workers within the IMSS. The occupation with the greatest representation was nursing staff, with 39.9% (93 nurses), followed by medical staff, with 34.8% (81 doctors).

Regarding the sociodemographic variables, 53.6% (125 families) have a simple nuclear family (this means that it was composed of a mother, father, and one to three children), and 66.5% (155 families) have an integrated nucleus (this means that all family members live in the same house). The number of children per worker was 1.69 ± 1.3 (68.7%, 160 workers) had at least a bachelor's degree, and 62.7% (146 workers) had a stable partner (couples with and without civil union). The average body mass index (BMI) of the workers was 27.7 ± 4.2 kg/m^2^. Regarding physical activity, 29.2% (68 workers) were sedentary, and 51.9% (121) did mild exercise. Smoking was at 18.5% (43 workers), and frequent alcohol consumption was at 21.5% (50 workers).

The mean score of the instruments was 12.93 ± 10.2 for HAS, 8.08 ± 7.4 for BDI, 7.37 ± 4.9 for PSQI, and 8.9 ± 1.9 for GMC-SLE. When stratified, it implied that 75.6% (176 workers) had some degree of anxiety, 18.9% (44 workers) had some degree of depression, and 56.7% (132 workers) had poor sleep quality. As for the MBOI, the alteration of only one domain of the instrument occurred in 32.6% (76 workers), the two-domain alteration occurred in 26.2% (61 workers), and 17.2% (40 workers) had three domains of the inventory affected.

Table 1 shows the sociodemographic, clinical, and family composition and the categorized results of the inventories used, compared according to the quality of the workers' sleep. Regarding the categories associated with poor sleep quality, those with a statistical difference were the work category (medical personnel), education (highest educational level), some degree of depression or anxiety, frequent alcohol consumption, and socioeconomic level (lower stratum), all with p < 0.01.

**Table 1:** Demographic and occupational characteristics of healthcare staff classified by sleep quality Abbreviations: Nu: nuclear, Ex: extensive, Dom: domain The chi-square test was used to compare the variables. *p value less than 0.05

Characteristic	Pittsburgh Sleep Quality Index			Characteristic	Pittsburgh Sleep Quality Index		
	Good Quality	Poor Quality	p value		Good Quality	Poor Quality	p value
	(≤5 points)	(≥6 points)			(≤5 points)	(≥6 points)	
	n (%)	n (%)			n (%)	n (%)	
All	101 (100)	132 (100)	-	All	101 (100)	132 (100)	-
Gender				Smoking			
Male	44 (43.56)	62 (46.97)	0.61	Yes	20 (19.8)	23 (17.47)	0.64
Female	57 (56.44)	70 (53.03)		No	81 (80.2)	109 (82.53)	
Type of contract				Frequent alcohol consumption			
Permanent workers	96 (95)	117 (88.7)	0.08	Yes	16 (15.84)	34 (25.76)	0.06
Non-permanent workers	5 (5)	15 (11.3)		No	85 (84.16)	98 (74.24)	
Category				Physical activity			
Doctor	24 (23.8)	57 (43.2)	<0.01*	Sedentary	29 (29)	39 (29.6)	0.69
Nurse	50 (49.5)	43 (32.6)		Mild	56 (56)	65 (49.2)	
Non-clinical	27 (26.7)	32 (24.2)		Moderate	13 (13)	24 (18.2)	
Education level				Intense	3 (3)	4 (3)	
Elementary school or less	1 (1)	1 (0.76)	0.04*	Marital status			
Middle school	0 (0)	2 (1.52)		Single	21 (21)	32 (24.2)	0.11
High school	22 (22)	25 (18.94)		Married without civil union	24 (24)	23 (17.4)	
Bachelor`s degree	56 (56)	53 (40.15)		Married with civil union	38 (38)	61 (46.2)	
Master’s degree or more	22 (22)	51 (38.63)		Divorced	18 (18)	13 (9.9)	
Previous illness or special health condition				Widowed	0 (0)	3 (2.3)	
Pregnancy and puerperium	1 (1)	3 (2.3)	0.57	Family relationship			
Diabetes mellitus	7 (7)	3 (2.3)		Nuclear (childless)	8 (8)	11 (8.3)	0.74
Diabetes and other	3 (3)	4 (3)		Simple Nuclear	58 (58)	67 (50.8)	
Arterial hypertension	8 (8)	15 (11.4)		Numerous Nuuclear	6 (6)	11 (8.3)	
Arterial hypertension and other (non-diabetes)	1 (1)	3 (2.3)		e.g., Extensive	4 (4)	7 (5.3)	
Other	17 (17)	20 (15.2)		e.g., Compound	2 (2)	0 (0)	
None	64 (64)	84 (63.5)		Nonparental	15 (15)	23 (17.4)	
Physical presence of the family in the home				Other	8 (8)	13 (9.9)	
Integrated nuclear	63 (63)	92 (70)	0.62	Hamilton Anxiety Scale (final classification)			
Non-integrated nuclear	21 (21)	27 (20.5)		No anxiety (0-5 points)	26 (25.7)	31 (23.5)	<0.01*
e.g., Descending	6 (6)	5 (3.8)		Minor anxiety (6-14 points)	57 (56.5)	41 (31)	
e.g., Ascending	7 (7)	5 (3.8)		Major Anxiety (≥ 15 points)	18 (17.8)	60 (45.5)	
e.g., Collateral	4 (4)	3 (1.9)		Graffar-Méndez-Castellanos socioeconomic level scale			
Beck Depression Inventory (final classification)				High stratum (4-6 points)	3 (3)	13 (9.9)	<0.01*
No depression (0-13 points)	93 (93)	96 (72.7)	<0.01*	Upper middle stratum (7-9 points)	57 (56.4)	72 (54.5)	
Mild depression (14-19 points)	5 (5)	19 (14.4)		Lower middle stratum (10-12 points)	41 (40.6)	35 (26.5)	
Moderate depression (20-28 points)	2 (2)	15 (11.4)		Working class (13-16 points)	0 (0)	12 (9.1)	
Severe depression (≥ 29 points)	1 (1)	2 (1.5)		Maslach Burnout Inventory (depersonalization domain)			
Maslach Burnout Inventory (emotional exhaustion domain)				With suspicion	57 (56.4)	78 (59)	0.68
With suspicion	64 (63.4)	75 (56.8)	0.31	Without suspicion	44 (43.6)	54 (41)	
Without suspicion	37 (36.6)	57 (43.2)		Maslach Burnout Inventory (final classification)			
Maslach Burnout Inventory (personal fulfillment domain)				Without suspicion	22 (21.8)	34 (25.7)	0.78
With suspicion	42 (41.6)	65 (49.2)	0.24	Burnout (1 dom.)	36 (35.6)	40 (30.3)	
Without suspicion	59 (58.4)	67 (50.8)		Burnout (2 dom.)	25 (24.8)	36 (27.3)	
-				Burnout (3 dom.)	18 (17.8)	22 (16.7)	

Regarding the continuous variables, those with a difference between sleep quality groups were the scores by domain (psychic and somatic) and total of the HAS instrument, the score of the BDI instrument, the score of the personal fulfillment domain of the MBOI, and hours of sleep (all with p < 0.01). The rest of these variables can be seen in Table 2.

**Table 2:** Comparison of age, job tenure, and anxiety, classified by quality of sleep Abbreviations: SD: standard deviation IQR: interquartile range The two-tailed student t-test or U-Mann Whitney test was used to compare independent variables. *p value less than 0.05

Characteristic	Good Quality (≤5 points)	Poor Quality (≥6 points)	p value
	Mean ± SD	Mean ± SD	
Age (years)	40 ± 8.7	41 ± 8.5	0.21
Number of children	1.8 ± 1.4	1.5 ± 1.2	0.1
Body mass index	27.35 ± 3.9	28 ± 4.4	0.2
Maslach Burnout Inventory score (emotional exhaustion domain)	21.4 ± 13	23.6 ± 13.1	0.22
Maslach Burnout Inventory score (depersonalization domain)	9.6 ± 6.6	8.8 ± 7.5	0.38
Maslach Burnout Inventory score (personal fulfillment domain)	30.3 ± 9.3	32.4 ± 8.9	0.04*
-	Median (IQR)	Median (IQR)	-
Pittsburgh Sleep Quality Index total score	3 (2)	10 (6)	<0.01*
Seniority in the IMSS	13 (15)	11 (14)	0.63
Seniority in the UMF	6 (8)	2 (8)	0.33
Hamilton Anxiety Scale score (physical domain)	4 (5)	8 (9)	<0.01*
Hamilton Anxiety Scale score (somatic domain)	3 (3)	5 (6)	<0.01*
Hamilton Anxiety Scale total score	8 (8)	13 (16)	<0.01*
Beck Depression Inventory total score	3 (6)	9 (9)	<0.01*
Hours in bed	8 (1)	7.75 (1.8)	0.95
Hours of sleep	7 (2)	6 (2)	<0.01*
Graffar-Méndez-Castellanos socioeconomic level scale (total score)	9 (2)	9 (3)	0.14

In the correlation matrix with the PSQI score, they have presented correlations with the HAS score in the psychological domain score (S = 0.30, p < 0.01), somatic domain score (S = 0.24, p < 0.01), and the total score (S = +0.30, p < 0.01), as well as with the MBOI in the personal fulfillment domain score (S = 0.13, p = 0.045), with the total BDI score (S = 0.50, p < 0.01), and with hours of sleep (S = 0.46, p < 0.01).

The SARS-CoV-2 emergency caused fear and uncertainty in the general population, particularly among healthcare workers, who were one of the groups most mentally affected [23]. In this regard, the survey of knowledge, attitudes, fears, and needs regarding COVID-19 in this research yielded interesting results to note. Health workers identified with poor sleep quality perceive that their family and friends avoid them for fear that they are a vector of COVID-19 because of their work (p = < 0.01). Furthermore, those with poor sleep quality think that if they get sick, they will suffer from severe forms of the disease (p = < 0.01). Unfortunately, the proportion of those who believe that the medical unit where they work has the necessary supplies and resources to treat cases of COVID-19 was low in those with poor sleep quality (p < 0.01). The rest of the items are contained in Table 3. The survey items were contrasted by sex, marital status, and job category; only the latter had significant differences. Non-clinical personnel are the ones who, to a greater extent, consider that they do not have sufficient information about the treatment and preventive measures of COVID-19. In addition, this same area considers that the information that the institution has provided has not been sufficient or clear and considers that the disease is difficult to treat, and, finally, the non-clinical staff believed that the unit does not have the necessary resources and supplies to treat the disease (all with p < 0.01).

**Table 3:** Sleep quality distributed by questions asked to staff and the comparison test Abbreviations: 1: Totally disagree + partially disagree + neither agree nor disagree, 2: partially agree + totally agree *p value less than 0.05. All hypothesis tests were two-tailed. The comparison of proportions was carried out using the chi-square test.

Characteristics	Pittsburgh Sleep Quality Index	
	Good Quality (≤5 points)	Poor Quality (≥6 points)
	n (%)	n (%)
All	101 (100)	132 (100)
I have sufficient information about the signs and symptoms of COVID-19.		
1	8 (7.9)	9 (6.8)
2	93 (92.1)	123 (93.2)
p value	0.75	
I have sufficient information about the prognosis of COVID-19.		
1	9 (7.32)	10 (7.6)
2	92 (92.68)	122 (92.4)
p value	0.71	
I have sufficient information about the treatment of COVID-19.		
1	13 (12.9)	16 (12.1)
2	88 (87.1)	116 (87.9)
p value	0.86	
I have sufficient information about the routes of transmission of COVID-19.		
1	14 (13.9)	11 (8.3)
2	87 (86.1)	121 (91.7)
p value	0.17	
I have sufficient information about precautionary measures for COVID-19.		
1	14 (13.9)	10 (7.6)
2	87 (86.1)	122 (92.4)
p value	0.12	
The IMSS has provided me with sufficient information about COVID-19.		
1	16 (15.8)	21 (15.9)
2	85 (84.2)	111 (84.1)
p value	0.98	
The information provided by the IMSS about COVID-19 has been clear.		
1	17 (16.8)	21 (15.9)
2	84 (83.2)	111 (84.1)
p value	0.85	
The information provided by the IMSS about COVID-19 has been sufficient.		
1	24 (23.8)	37 (28)
2	77 (76.2)	95 (72)
p value	0.46	
I do not require more information about COVID-19.		
1	24 (23.8)	37 (28)
2	77 (76.2)	95 (72)
p value	0.19	
I restrict my social contact because I consider myself dangerous.		
1	16 (15.8)	30 (22.7)
2	85 (84.2)	102 (77.3)
p value	0.19	
I feel like my family and friends avoid me because of my job.		
1	61 (60.4)	55 (41.7)
2	40 (39.6)	77 (58.3)
p value	<0.01*	
I am afraid of getting sick from COVID-19 and have looked for excuses not to go to work.		
1	52 (51.5)	82 (62.1)
2	49 (48.5)	50 (37.9)
p value	0.1	
If cases increase, I will miss work.		
1	80 (79.2)	104 (78.8)
2	21 (20.8)	28 (21.2)
p value	0.93	
I am afraid of COVID-19.		
1	38 (37.6)	56 (42.4)
2	63 (62.4)	76 (57.6)
p value	0.46	
If I get sick with COVID-19 I will have a serious form of the disease.		
1	37 (36.6)	66 (50)
2	64 (63.4)	66 (50)
p value	0.04*	
I am more susceptible to having a COVID-19 sequel.		
1	40 (39.6)	61 (46.2)
2	61 (60.4)	71 (53.8)
p value	0.31	
COVID-19 is difficult to treat.		
1	27 (26.7)	43 (32.6)
2	74 (73.3)	89 (67.4)
p value	0.34	
I think the UMF can deal with COVID-19.		
1	27 (26.7)	59 (44.7)
2	74 (73.3)	73 (55.3)
p value	<0.01*	
We should have more psychological support from the IMSS.		
1	13 (12.9)	22 (16.7)
2	88 (87.1)	110 (83.3)
p value	0.42	
What worries me most about COVID-19 is		
Isolation	3 (3)	2 (1.5)
Risk of infecting my family	74 (73.3)	101 (76.5)
Risk of becoming seriously ill	8 (7.9)	14 (10.6)
Not counting skills to treat the disease	16 (15.8)	15 (11.4)
p value	0.58	

According to the binary logistic regression model (Table 4), the risk factors identified for poor sleep quality were a higher BDI score (OR: 1.21, CI 95%: 1.13-1.29), being a doctor (OR: 3.48, CI 95%: 1.5-8.01), frequent alcohol consumption (OR: 2.4, CI 95%: 1.13-5.2), and, as a protective factor, a lower score in the depersonalization domain of the MBOI (OR: 0.5, CI 95%: 0.26-0.99).

**Table 4:** Binary logistic regression model (independent variable poor sleep quality: PSQI score ≥ 6 points) Cox y Snell’s R^2^: 0.24, Nagelkerke’s R^2^: 0.32 *p value less than 0.05

Characteristics	B	p value	OR	CI 95%	
				Lower	Upper
Maslach Burnout Inventory score (depersonalization domain)	-0.689	0.04*	0.504	0.26	0.99
Beck Depression Inventory total score	0.19	<0.01*	1.21	1.13	1.29
Category (doctor)	1.247	<0.01*	3.48	1.5	8.01
Category (nurse)	0.211	0.59	1.23	0.56	2.7
Frequent alcohol consumption	0.885	0.02*	2.4	1.13	5.2
Some degree of anxiety (Hamilton Anxiety Scale score ≥6 points)	-0.404	0.279	0.67	0.321	1.39

## Discussion

In the context of the SARS-CoV-2 pandemic, the results of this study indicate that healthcare workers were highly likely to have poor sleep quality (56.7%, 132 workers) and some degree of anxiety (75.6%, 176 workers). This particularly high magnitude was also documented in other countries during the epidemic; for example, a study conducted in 2020 in the Kingdom of Bahrain found higher prevalences of poor sleep quality and anxiety (75% and 84%, respectively) among health workers [24]. In the Mexican population, an investigation conducted in 2023 identified a prevalence of poor sleep quality of 52%, lower than that reported in this study [25]. Another study conducted in 2021 in Brazil found a prevalence of poor sleep quality of 73.4% with a significant relationship (p < 0.001) between symptoms of anxiety, depression, and insomnia, as in our study, where a relationship was identified between some degree of anxiety and depression (both with p < 0.01) and poor sleep quality [26].

Regarding the PSQI score, a study conducted in 2020 among healthcare workers in Spain found a mean of 8.78 ± 4.5 higher than that found in the present study (7.37 ± 4.9). No differences were identified between sex, worker category, or age, the latter being significant for the first level unit investigated (both with p < 0.05). No differences were found by sex, marital status, or work category, with medical personnel presenting a lower PSQI score. Although the personnel was not found in direct contact with COVID-19 patients, they had a lower score, but this difference was not significant [27]. These points differed from what was identified since the medical personnel presented a higher proportion of poor sleep quality and greater affection than the clinical personnel, with a significant difference (p < 0.01).

Under the same Mexican population context, but in a second-level unit, Garduño-Alanis et al. in 2023 identified a prevalence of 52% of poor sleep quality, with a strong association with depression and anxiety (18.7% and 29.4% in those with poor sleep quality), and even depression was considered an important risk factor for poor sleep quality with an OR of 7.23 (CI 95%: 1.85-28.14) [25]. In contrast to the present study, the prevalence was lower (18.9%), although the associations persisted, as major anxiety (45.5%) and depression (27.7%) had a higher prevalence in those with poor sleep quality, being that a higher score on the IDB was considered a risk factor for poor sleep quality with an OR of 1.21 (CI 95%: 1.13-1.29).

Returning to the study of sleep quality in the context of COVID-19 in Poland, Krupa et al. [28] identified sleep impairment in healthcare workers during the pandemic as high as 40%, in contrast to our study population, which was higher (56.7%), with insomnia being the most frequent (44.5%) and strongly associated with age and nursing clinical staff. The greater severity of insomnia was significantly higher in nursing staff (p < 0.01), in those who had more possibility of contact with COVID-19, and in those who did not know if they could be in contact with patients with COVID-19 (p < 0.05). The sleep quality of the nursing staff was the most affected compared to other types of staff in our study (p < 0.01) as well as what was found by Krupa et al.

In Egypt (2020), 83.1% of healthcare workers were found to be afraid of getting sick [29]. In Belgium, in the first year of the pandemic (2020), the prevalence of healthcare workers reporting feelings of fear ranged from "slightly afraid to moderately afraid" due to COVID-19 in 41% of healthcare workers. Some factors associated with this prevalence were female gender, lack of personal protective equipment (PPE), and inadequate education about COVID-19 [30]. In our study, more than half (59.65%) of the clinical and non-clinical staff reported fear of becoming ill with COVID-19, of which 62% reported poor sleep quality. The availability of CPSD played an important role, during the pandemic, in the mental health of health personnel as never before, which increased due to the scarcity of CPSD worldwide; in the same study cited above, 57.83% of the CPSD was available at the end of follow-up, very similar to our study population, where 63% considered that the medical unit had the necessary supplies for COVID-19 care [30].

Mental health and the safety of the functions performed by health personnel were related to information acquired about COVID-19 disease, which impacted the safety of healthcare actions. This is important because only 51.99% considered the information acquired about COVID-19 sufficient. Studies in countries with different economic development as Gana found that lower education about COVID-19 is a factor that influences the psychological deficiencies of healthcare workers [31]. Our population behaves differently and, at the same time, in a contradictory way because the poor quality of sleep was not associated with the information received about COVID-19. This behavior could be justified because if the person is better informed, he or she will be more careful and thus less afraid of getting sick. At the same time, there is no justification for not going to work, as in our study population.

Few studies consider burnout as one of the factors to be taken into account for sleep quality; what was interesting among those identified was the relationship with the MBOI score, where a higher score in the dimension of depersonalization was associated with poor sleep quality, probably due to the increase in self-demanding workers, which in turn can be associated with higher levels of anxiety. In addition, there is no in-depth research in the scientific literature on the opinion of workers regarding their attitudes and fears concerning COVID-19, finding, in our work, the thought (very possibly true) about the isolation caused by the fear of the population of health unit workers, and the fact of avoiding going to work because of COVID-19, especially the workers' fear of infecting their family. Furthermore, the depersonalization of health workers directly affects the ability to empathize with patients, showing the health worker cold, distant, and disinterested in the patient's health, causing a bad experience for the latter and a great barrier to the adequate doctor-patient relationship.

What is undeniable is that more than half of the workers surveyed in the UMF have poor sleep quality (56.7%, 132 workers), and this is related to anxiety, depression, and alcohol consumption. Because this is a topic little explored by health institutions and because of the type of design used in this research, it is not possible to know if these figures have increased during the pandemic or the directionality of the causality between the associated factors. However, they are the first diagnosis of the mental health of workers and set the tone for future research, being the follow-up and intervention in favor of the mental health of workers fundamental issues to explore. This research represents a starting point to delve deeply into the mental health of workers, allowing the health institution to develop evidence-based strategies for the timely identification of high-risk patients for poor sleep quality, which, if treated appropriately, would reduce the risk of workers suffering from the complications of said alteration.

Among the limitations and possible biases in this research, one of the most relevant is the possibility of hiding part of the information, as these are sensitive topics on the part of the workers, who may have felt compelled to lie about their answers. They sought to avoid this by providing a comfortable and safe space to answer the instrument and by showing absolute respect for the worker's reliability. Another possibility is selection bias since, although all workers who met the selection criteria were included, there may be self-selection on their part due to a higher (or lower) degree of anxiety, depression, or poor sleep quality; however, 100% acceptance was achieved for those who met the selection criteria.

## Conclusions

More than half of the workers at primary care units (56.7%, 132 workers) experience poor sleep quality. This is an important issue to address since, in Mexico, these units must resolve more than 80% of the reasons for the consultation of users. The effects of poor sleep quality are reflected in increased staff illnesses and a reduced ability to make decisions, directly impacting workers' awareness. The methodology of the present study makes it difficult to know whether or not the COVID-19 pandemic increased the prevalence of poor sleep quality in workers. This study does allow us to identify the association and the greater risk of suffering from poor sleep quality in workers who suffer burnout (especially with depersonalization), who present some degree of depression, who are part of the medical and nursing staff, and, who consume alcohol with frequency. There are fundamental bases to establish guidelines for the diagnosis and identification of personnel with a risk approach since workers are the most important capital of health institutions.

The mental health of workers should be a central point of health institutions, it has been neglected for a long time. Strategies must be established in public policy to protect it. The only way to be sure of its beneficial effect is to measure it before applying it and afterward to know its effect. This is the best way to create evidence-based public policy.
